# Effect of Feed Restriction on the Behaviour and Welfare of Broiler Chickens

**DOI:** 10.3390/ani10050830

**Published:** 2020-05-11

**Authors:** Angela Trocino, Peter White, Francesco Bordignon, Valentina Ferrante, Daniela Bertotto, Marco Birolo, Giulio Pillan, Gerolamo Xiccato

**Affiliations:** 1 Department of Comparative Biomedicine and Food Science (BCA), University of Padova, Viale dell’Università 16, 35020 Legnaro, Padova, Italy; francesco.bordignon.3@phd.unipd.it (F.B.); daniela.bertotto@unipd.it (D.B.); giulio.pillan@phd.unipd.it (G.P.); 2 Sydney School of Veterinary Science, Faculty of Science, The University of Sydney, B19 R.M.C. Gunn Building, Sydney, NSW 2006, Australia; p.white@sydney.edu.au; 3 Sydney Institute of Agriculture, The University of Sydney, B19 R.M.C. Gunn Building, Sydney, NSW 2006, Australia; 4 Institute of Animal Science and Technology, Group of Aquaculture and Biodiversity, Polytechnic University of Valencia, Camino de Vera 14, 46022 Valencia, Spain; 5 Department of Environmental Science and Policy, University of Milan, Via Celoria 10, 20133 Milan, Italy; valentina.ferrante@unimi.it; 6 Department of Agronomy Food Natural resources Animal and Environment (DAFNAE), University of Padova, Viale dell’Università 16, 35020 Legnaro, Padova, Italy; marco.birolo@unipd.it (M.B.); gerolamo.xiccato@unipd.it (G.X.)

**Keywords:** genotype, sex, feed restriction, age, poultry

## Abstract

**Simple Summary:**

Commercial genotypes of broiler chickens reared for meat purposes in intensive rearing systems are selected for fast growth and high breast yield and kept under conditions that may negatively affect normal behavioural patterns and reduce activity levels. Besides, quantitative feed restriction, used in the field to manipulate chicken growth and improve feed efficiency, may produce hunger and abnormal behaviours. Under these conditions, health and welfare are at risk. Thus, the present study aimed to evaluate the effect of genotype, sex and feed restriction on the welfare of broiler chickens by examining behaviour and corticosterone as a stress indicator during the production cycle. Results showed that behavioural differences between two fast-growing genotypes were limited in quantity and quality, whereas sex and age played a major role. The effect of sex was related especially to size so that heavier males were more active at earlier ages but moved less towards the end of the trial when their walking ability/comfort decreased. Feed restriction stimulated activity during and beyond the restriction phase without relevant effect on stress condition as measured by the plasma and faeces corticosterone.

**Abstract:**

Under intensive rearing conditions, the welfare of broiler chickens may be at risk depending on genotype and sex, due to their different growth rates. The practice of quantitative feed restriction may also impact on welfare. This study aimed to evaluate behaviour and corticosterone content in plasma and faeces at different ages using 896 one-day-old chicks housed in 32 pens, allocated to 8 groups, i.e., 2 genotypes (standard vs. high breast yield) × 2 sex × 2 feeding plans (ad libitum vs. restricted, AL vs. FR). The feeding system affected the percentage of standing (9.84% vs. 11.7% in AL vs. FR; *p* ≤ 0.001), feeding (7.51% vs. 8.17%; *p* ≤ 0.01) and sitting/lying (67.0% vs. 64.1%; *p* ≤ 0.001), and the faeces corticosterone content (12.2 vs. 13.6 ng/g in AL vs. FR; *p* ≤ 0.10). Sex affected the percentage of pecking other chickens, standing and comfort behaviours. Changes in behaviour were recorded between high and standard breast yield genotypes with faeces corticosterone which tended to be higher in the former (*p* ≤ 0.10). Significant interactions between the main factors and age were observed. Major changes in behaviour were due to feed restriction, which stimulated activity during restriction.

## 1. Introduction

Commercial genotypes of broiler chickens reared for meat purposes in intensive rearing systems are selected for fast growth and high breast yield [1]. Under these intensive conditions, health and welfare are at risk. A high incidence of metabolic diseases, such as ascites, lameness, mortality and sudden death syndrome, may be recorded [2,3]. Besides, rearing conditions (high stocking density, poor litter quality) may negatively affect the occurrence of normal behavioural patterns due to low activity levels [4,5]. Both male and female chickens are affected, but likely in different ways. Since males grow faster and reach a higher body weight than females, some differences may occur in locomotory activity [6] and aggressive behaviours [7].

In the field, quantitative feed restriction is used to manipulate chicken’s growth and decrease growth rate with the final aim of limiting the occurrence of metabolic diseases [8] and improving feed efficiency. Despite these positive gains, feed restriction can compromise animal welfare. Feed restricted broilers may feel hunger and may show abnormal behaviours such as over-drinking, increased pacing, pecking at fixtures and increased aggression [9,10,11]. Useful information regarding the welfare of broilers may be derived from both behavioural and physiological indicators [12]. In the first case, ethograms can be used to highlight abnormal and stereotyped behaviours (indexes of poor welfare). In the second, stress level (affected by an unsuitable environment and social relationships) can be estimated from the corticosterone concentration measured in different mediums such as blood and faeces [13,14].

The present study aimed to evaluate the effect of genotype, sex and feed restriction on the welfare of broiler chickens by examining behaviour and corticosterone as a stress indicator at different ages during the production cycle.

## 2. Materials and Methods

The study was approved by the Ethical Committee for Animal Experimentation (Organismo per la Protezione del Benessere Animale, OPBA) of the University of Padova (project 34/2014; prot. N. 89574 of June 19, 2014). All animals were handled according to the principles stated by the EU Directive 2010/63/EU [15] regarding the protection of animals used for experimental and other scientific purposes. Research staff involved in animal handling were animal specialists (PhD or MS in Animal Science) and veterinary practitioners.

### 2.1. Housing

The trial was performed at the poultry house of the Experimental Farm “Toniolo” at the University of Padova (Legnaro, Padova, Italy). The poultry house was equipped with a cooling system, forced ventilation, radiant heating and controlled light systems. Thirty-two wire-net pens (125 cm width × 177 cm length × 120 cm height; 2.2 m^2^) were available, each equipped with an automatic circular drinker (diameter: 39 cm) and a circular feeder (diameter: 37 cm) for manual distribution of feed. The pens had a concrete floor bedded with wood shavings as litter (height 5 cm, 2.5 kg/m^2^). Twenty-four hours of light were provided during the first two days following the arrival of chicks. Lighting was progressively reduced to maintain a ratio of 18 h of light and 6 continuous hours of dark from the 12th day onwards.

### 2.2. Animals and Experimental Groups

A total of 896 one-day-old chicks were used for evaluating behaviour and physiological indicators of stress as reported in this paper. Growth performance, carcass and meat quality, occurrence of white striping and wooden breast at gross examination and the evolution of muscle fibre degeneration at different ages were also measured in the same trial and results are given in other papers [16,17].

All chicks were delivered by authorized transport means to the experimental facilities of the University of Padova on the day of hatching. Half chicks were high breast yield genotype (Ross 708) and the other half of a standard breast yield type (Ross 308); within genotype, all chicks were sexed at the hatchery. On arrival, 28 chicks were housed in each of 32 pens, randomly allocated to 8 experimental groups, i.e., 2 genotypes × 2 sex × 2 feeding plans (ad libitum vs. restricted) and controlled from the day after their arrival until commercial slaughtering at day 46. Final stocking density was 24 chickens per pen (i.e., approximately 11 chickens/m^2^). During the experimental trial, half the pens were fed ad libitum (AL group), the remaining half were fed a restricted diet between days 13 to 21 (FR group). The restriction period and length were chosen on the basis of previous literature to exploit the expected compensatory growth of chickens [16]. The FR groups received 80% of the quantity consumed by the chickens fed the ad libitum diet on the previous day. The restriction program was calculated separately for the four groups obtained by the combination of 2 genotypes × 2 sex. Four commercial diets were administered during the trial, i.e., diet P1 (crude protein 22.2%, ether extract 7.90%; crude fiber 2.60%, calcium 1.00%, and phosphorus 0.70%) from 0 to 12 d, diet P2 (crude protein 20.8%, ether extract 8.50%; crude fiber 2.50%, calcium 1.00%, and phosphorus 0.65%) from 13 to 21 d, diet P3 (crude protein 19.0%, ether extract 8.10%; crude fiber 2.50%, calcium 0.95%, and phosphorus 0.60%) from 22 to 35 d and diet P4 (crude protein 17.4%, ether extract 8.80%; crude fiber 2.40%, calcium 0.80%, and phosphorus 0.60%) from 35 d until slaughtering. Further details about the experimental facilities, the management of the chickens and the procedures of commercial slaughter are given by Trocino et al. [16].

### 2.3. Behavioural Observations

Behaviour was recorded at the pen level by video-recording for 24 h on 16 pens (2 per experimental group) at different ages, i.e., days 11, 18, 25, 32, 39 and 45. Then, the number of chickens per pen performing predetermined behaviours were scored every 30 minutes throughout the light period by scanning 10 consecutive seconds of video by one observer. Behaviours were selected based on Nielsen et al. [18], as reported in Table 1, and were mutually exclusive.

Specific observations focused on the behaviour of animals on the same 16 pens at day 18 during feed restriction and near the time of feed administration. In detail, 10 consecutive seconds were scanned 10 min before the feed administration, during the feed administration, and then 5, 10, and 15 min after the feed administration. In the case of feed-restricted chickens, feed administration matched with the moment when feeders were filled with feed and put into the pen. In the case of chickens fed ad libitum, the feed administration matched with the moment when feeders were removed from the pen, weighed, filled with the diet and placed back into the pen.

### 2.4. Sampling and Analysis for Corticosterone in Plasma and Faeces

On days 14, 21, 28 and 35, 32 chickens (one chicken per pen) were slaughtered by cervical dislocation to sample muscles for histological analyses [17]. The same animals were used for sampling plasma from blood immediately after slaughter. At the same time, faeces were sampled as a pool from each pen collecting them from the litter. Corticosterone levels in plasma and faeces were measured by a specific microtiter radioimmunoassay (RIA) [19] using a rabbit anti-corticosterone serum (Biogenesis, Poole, UK). Before RIA, steroids were extracted with 8 mL of diethyl ether from plasma (100 μL), or from faeces (50 mg) that were homogenized and pulverized in liquid nitrogen and the homogenate was then recovered with 1 mL of sodium citrate buffer hydrolysed with β-glucuronidase and sulfatase (SIGMA from *Helix pomatia*) for one hour at 37 °C. Dry extracts were then dissolved in 1 mL of phosphate buffer (PBS, pH 7.2) and various aliquots were used for the assay depending on the steroid content in samples. The anti-corticosterone serum showed the following cross-reactions: corticosterone 100%, 11-deoxycorticosterone 1%, cortisol 0.1%, progesterone 0.02%, testosterone < 0.01%. The sensitivity of the assay was 1.5 pg/well and was defined as the dose of the hormone at 90% binding. To validate RIA corticosterone measurements in plasma and faeces, parallelism and intra- and inter-assay tests were performed. The displacement curves of the diluted extracts were parallel to the standard curve in both the matrices. The corticosterone assay exhibited very good precision and reproducibility showing intra-assay and inter-assay coefficients of variation lower than 2% and 4% respectively, both in plasma and faeces. The mean recovery rates of corticosterone added to plasma and faeces were 79% and 65%, respectively.

### 2.5. Statistical Analysis

Behavioural data (as a percentage of animals performing a behaviour in each pen per scan) were subjected to analysis of variance by using a mixed model and the PROC GLIMMIX of SAS [20], with genotype, sex, feeding system, animal age, and their interactions as fixed effects and hour as a random effect. Data from the same pen were treated as repeated measures. A Poisson distribution was assumed for these data. The probability values of all interactions are reported in Table S1.

Behavioural data focused on the restriction feeding week (day 18) were also analysed by a mixed model and the PROC GLIMMIX [20] with genotype, sex, feeding system, time with respect to feed administration and their interactions as fixed effects and pen as a random effect. The probability values of all main factors and all interactions are reported in Table S2.

Finally, data for corticosterone levels in faeces and plasma were analysed by PROC MIXED [20] with genotype, sex, feeding system, animal age and their interactions as main factors and pen as a random effect. Differences among means with *p* < 0.05 were accepted as being statistically significant.

## 3. Results

### 3.1. Behavioural Observations During the Trial

#### 3.1.1. Effect of Age

**Figure 1:**
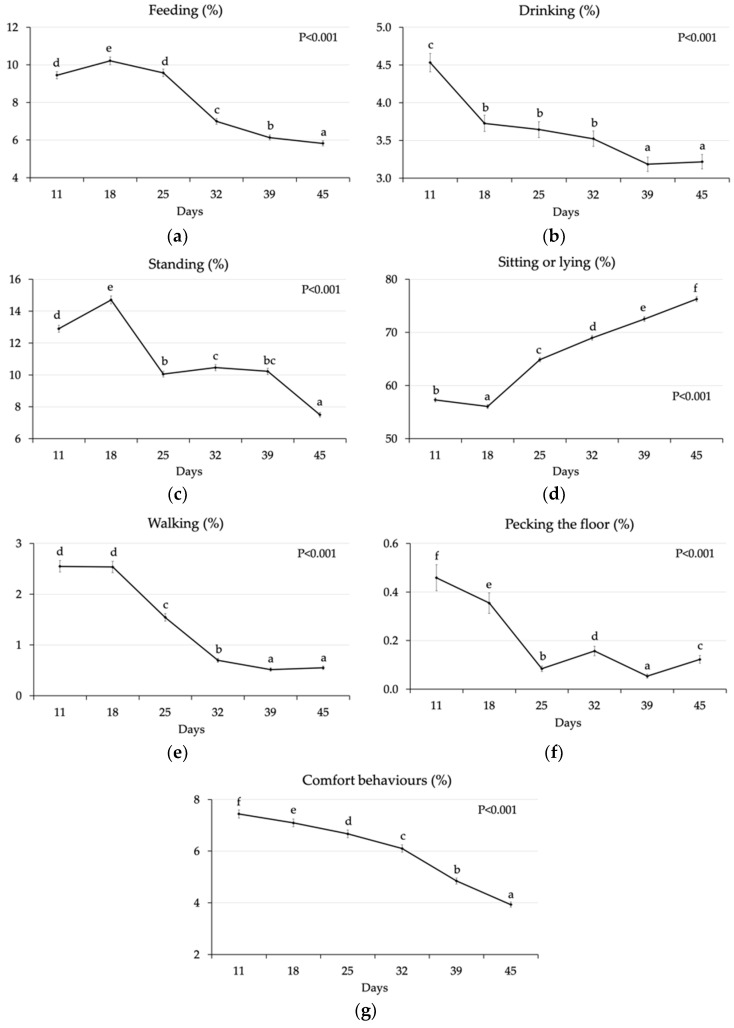
Percentage of chickens per pen (LS means ± SEM) feeding (**a**), drinking (**b**), standing (**c**), sitting or lying (**d**), walking (**e**), pecking the floor (**f**) and performing comfort behaviours (**g**) at different ages. Different letters stand for significant differences among LS means (probability of the Bonferroni t-test).

The significant changes in chicken behaviour according to age are represented in Figure 1. The percentage of chickens feeding increased (9.45% to 10.2%, *p* < 0.001) from the first to the second recording (11 d to 18 d) and then decreased until the lowest value measured at 45 d of age (5.83%) (*p* < 0.001) (Figure 1a). The percentage of chickens at drinkers decreased from 11 to 18 d (4.53% *vs* 3.73%; *p* < 0.001), remaining stable until 32 d, and then decreased again from 32 d to 39 d (3.52% *vs* 3.19%) (Figure 1b). Standing bird percentage increased from 11 d to 18 d (12.9% vs. 14.7%), then decreased until 25 d (*p* < 0.001), to remain stable between 32 d and 39 d (Figure 1c). Finally, from 39 d to 45 d of age, the percentage of standing birds decreased (10.2% vs. 7.50%; *p* < 0.001). The percentage of birds sitting or lying increased from the recordings at 18 d (56.1%) to the following recordings and until 45 d of age (76.3%) (*p* < 0.001) (Figure 1e). Conversely, the percentage of chickens walking and pecking the floor decreased from the first two recordings at 11 d and 18 d to the recording at 25 d and 32 d, to remain lower than previous recordings and rather stable from 39 d to 45 d (Figure 1e,f). On the other hand, the percentage of chickens performing comfort activities showed a constant decrease from the first observation to the following and until the last one (Figure 1g).

#### 3.1.2. Effect of Feeding System, Genotype, and Sex

The average of the six recordings at different ages found the percentage of animals standing (11.7% vs. 9.84%; *p* < 0.001) or at feeders (8.17% vs. 7.51%; *p* = 0.01) higher in FR compared to AL chickens, whereas the percentage of chickens sitting/lying was lower in the former compared to the latter group (64.1% vs. 67.0%; *p* < 0.001) (Table 2). Genotype affected the percentage of chickens standing during observations, being lower in standard vs. high breast yield groups (*p* < 0.001), whereas other minor changes were not relevant from a quantitative and qualitative point of view. Regarding sex, a higher percentage of males was found pecking other chickens compared to females (0.11% vs. 0.05%; *p* < 0.001), whereas a lower percentage of males was observed standing (10.5% vs. 10.9%; *p* < 0.001) and in comfort behaviours (5.68% vs. 6.06%; *p* = 0.05), but changes were low in absolute values (Table 2).

**Figure 2:**
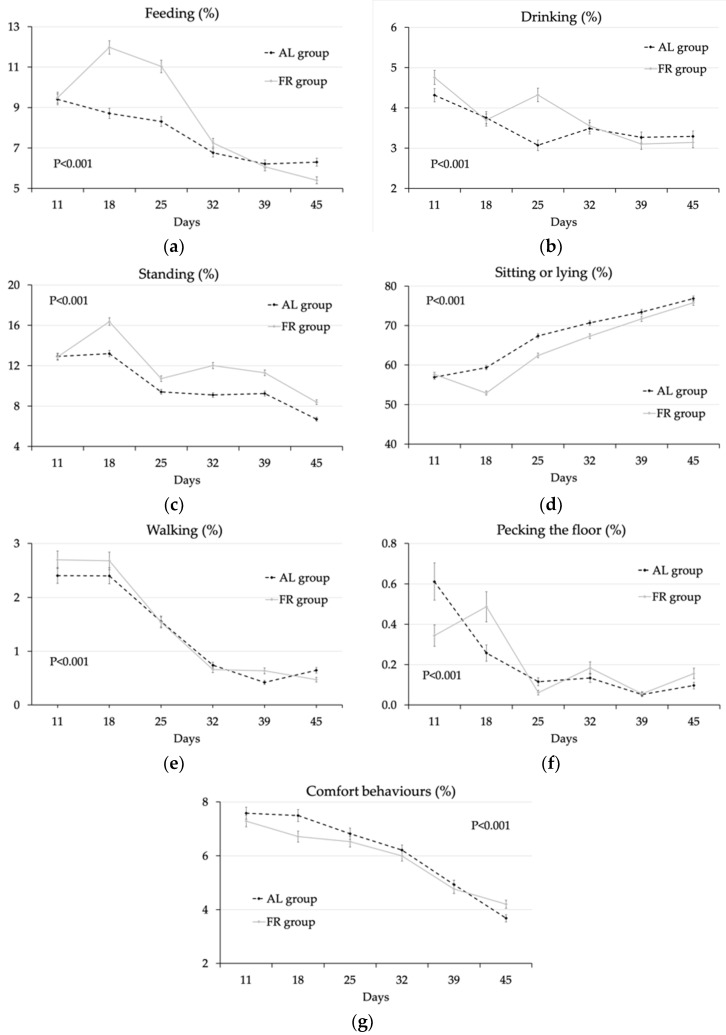
Percentage of chickens per pen (LS means ± SEM) fed ad libitum (AL, black dotted line) or submitted to restriction (FR, grey full line) feeding (**a**), drinking (**b**), standing (**c**), sitting or lying (**d**), walking (**e**), pecking the floor (**f**) and performing comfort behaviours (**g**) at different ages (Probability of the interaction feeding system × age, *p* < 0.001). LS means of AL chickens vs. FR chickens are compared within the same age.

Regarding the significant interactions between age and the other main factors (Table S1), a higher percentage of birds at feeders was recorded in the FR group especially at 18 d (12.0% vs. 8.71%) (when restriction started) and later, at 25 d (11.0% vs. 8.31%) (when the restriction period had ended) (significant probability of the interaction feeding system × age; *p* < 0.001) (Figure 2a). Only 10 d after the end of restriction feeding (32 d of age) the percentage of FR birds at feeders reached values similar to those fed ad libitum (7.01%, on average) (Figure 2a). The percentage of chickens at drinkers differed between groups only at 25 d (Figure 2b). On the other hand, the percentage of standing birds was lower in the FR than in the AL group from 18 d (feed restriction) until the end of the trial (Figure 2c). The percentage of sitting/lying birds showed an opposite pattern, even if differences between the two groups disappeared by 39 d of age (Figure 2d). Changes in walking were erratic (Figure 2e). During feed restriction (18 d), the percentage of birds pecking the floor (Figure 2f) was higher in the FR compared to the AL group (0.49% vs. 0.26%), whereas the percentage of birds performing comfort activity (Figure 2g) was lower (6.71% vs. 7.50%) (*p* < 0.001).

**Figure 3:**
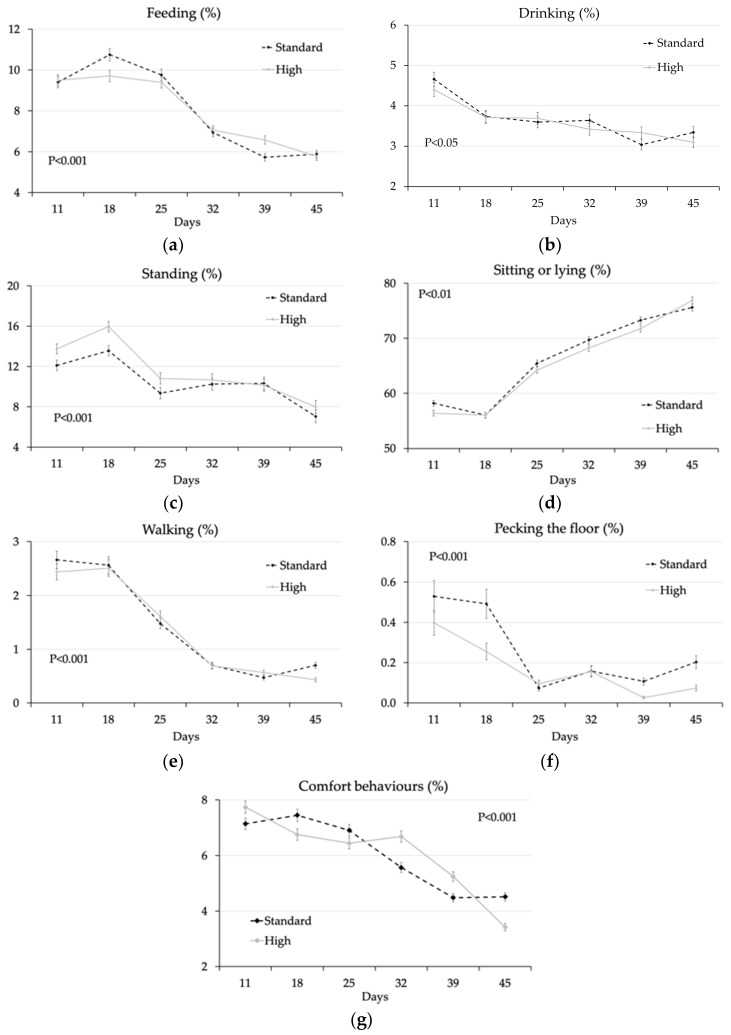
Percentage of chickens per pen (LS means ± SEM) of the standard-breast genotype (black dotted line) or the high-breast genotype (grey full line) feeding (**a**), drinking (**b**), standing (**c**), sitting or lying (**d**), walking (**e**), pecking the floor (**f**) and performing comfort behaviours (**g**) at different ages (probability of the interaction genotype × age, *p* < 0.001). LS means of the standard-breast vs. the high-breast genotype are compared within the same age.

With respect to genotype × age interactions (Figure 3), in the week of rationing (at 18 d) more standard yield chickens were observed at feeders (10.8% vs. 9.71%; *p* < 0.001) (Figure 3a) and pecking the floor (0.49% vs. 0.26%; *p* < 0.001) (Figure 3f) than high yield chickens, whereas at 39 d the percentage of high yield chickens at feeders was higher compared to standard yield ones (6.57% vs. 5.73%; *p* < 0.001). On the other hand, the percentage of standing birds was significantly higher in those with a high breast yield compared to those with a standard breast yield at 11 d (13.7% vs. 12.1%; *p* < 0.001) and 18 d (16.0% vs. 13.6%; *p* < 0.001), after which the behaviour of the two genotypes was similar (Figure 3c). Differences in birds sitting/lying (Figure 3d) were limited to the first week, whereas those in walking animals only related the last observation (Figure 3e). The percentage of birds drinking (Figure 3b) and engaged in comfort behaviours (Figure 3g) did not show a clear pattern at the different ages according to genotype.

**Figure 4:**
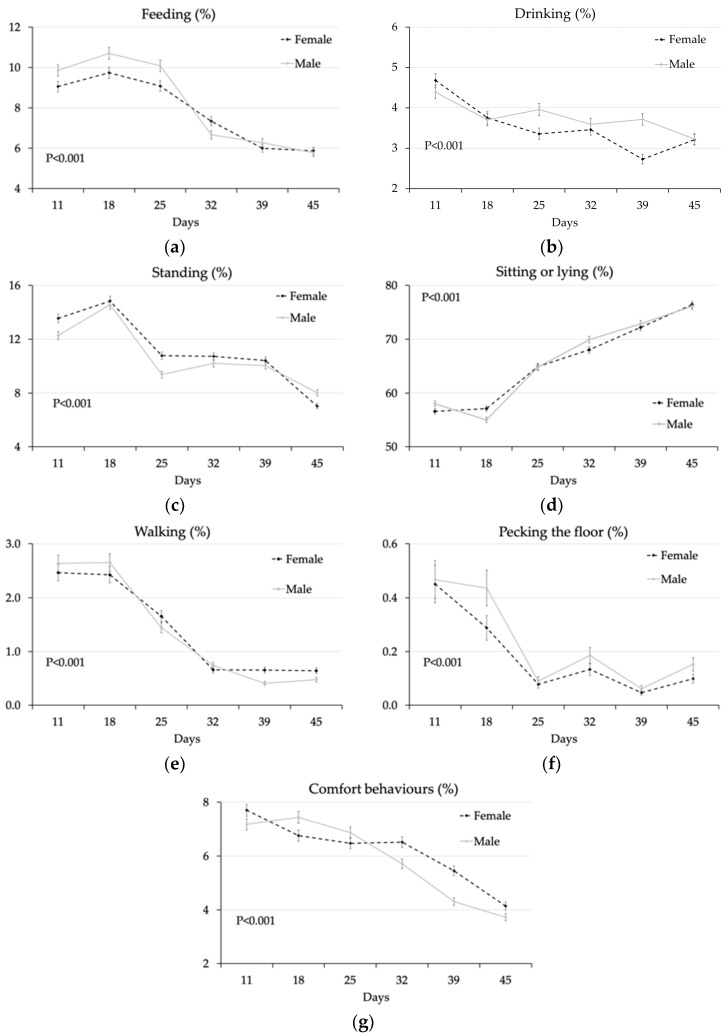
Percentage of chickens per pen (LS means ± SEM): females (black dotted line) or males (grey full line) feeding (**a**), drinking (**b**), standing (**c**), sitting or lying (**d**), walking (**e**), pecking the floor (**f**) and performing comfort behaviours (**g**) at different ages (Probability of the interaction sex × age, *p* < 0.001). LS means of females vs. males are compared within the same age.

In terms of the sex × age interaction (Figure 4), until 25 d the percentage of chickens feeding was significantly higher for males than for females (*p* < 0.001) and only later were differences reduced (Figure 4a), whereas an opposite pattern was observed for the percentage of chickens drinking (Figure 4b). On the other hand, the percentage of standing chickens was usually higher for females than males, but in the week of restriction feeding (18 d) and later at 32 and 39 d (Figure 4c). Differences in sitting/lying were small and related only to 18 d and 32 d of age (Figure 4d), whereas a higher percentage of walking females was recorded at the late observations at 39 d and 45 d of age (Figure 4e). A higher number of males was seen pecking the floor only at 28 d (Figure 4f). Finally, the percentage of chickens engaged in comfort behaviours took a different course in males and females during the first part of the trial compared to the second: the percentage of males in comfort behaviours was higher during restriction (7.44% vs. 6.76% *p* < 0.05) and lower from 32 d of age than that of females (Figure 4g).

### 3.2. Behavioural Observations during Feed Administration in the Restriction Period

Ten minutes before feeding, 2.80% of AL birds were at feeders (data not shown in the table). At the time of feed distribution, the percentage of birds at feeders was on average equal to 33.1%, remained stable at high values for the first 5 min (32.3%), and then gradually dropped (21.2% and 11.1%) 10 and 15 min after feed distribution. The percentage of standing (from 10.4% to 27.3%) and walking (from 0.83% to 6.72%) chickens increased significantly from 10 min before feed distribution and then decreased over the next 15 min (*p* < 0.001) (Table 3).

On the contrary, the percentage of chickens sitting or lying on the litter showed an opposite trend, decreasing from 74.4% (measured 10 min before feed distribution) to 8.87% and 7.33% (at distribution time and after 5 min), and then increasing gradually over the next 10 min (27.8% and 43.5%). The percentage of chickens at drinkers gradually increased from 0.84% before feed distribution to 6.97% at 15 min after feed administration. Chickens pecking other birds were observed only at the time of feed distribution whereas those performing comfort behaviour were only rarely observed. These latter data are not reported in tables as they were not suitable for statistical analysis.

**Figure 5:**
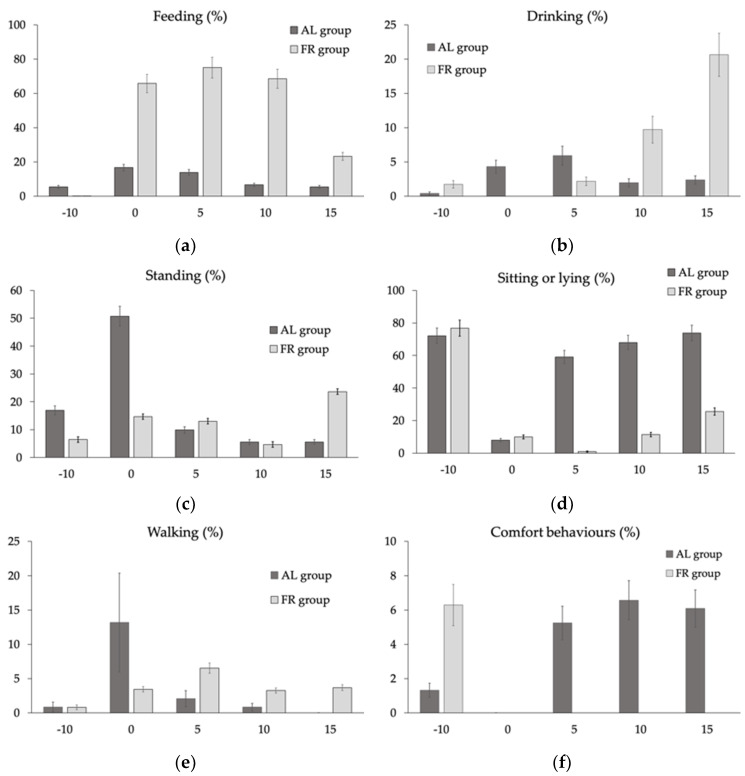
Percentage of chickens per pen (LS means ± SEM) fed ad libitum (AL) or submitted to restriction (FR) feeding (**a**), drinking (**b**), standing (**c**), sitting or lying (**d**), walking (**e**) and performing comfort behaviours (**f**) at different times with respect to feed distribution at 18 d (Probability of the interaction feeding system × time with respect to feed distribution, *p* < 0.001).

Regarding other factors (feeding system, genotype, sex) during the 25 min considered, the percentage of chickens sitting or lying changed according to the feeding system (on average 44.2% vs. 11.5% in AL vs. FR chickens; *p* < 0.001), whereas chickens standing changed with the genotype (on average 9.48% vs. 13.6% in standard vs. high breast yield) (data not reported in tables). On the other hand, behaviour was largely different depending on interactions of the main factors with time according to feed distribution (Table S2), as represented in Figure 5.

The percentage of AL chickens at feeders increased at the time of feed distribution and gradually decreased during the following 15 min, whereas the percentage of FR chickens at feeders remained high (>60%) from the time of feed distribution and for 10 min after, then showing a reduction to 23% at 15 min following feed distribution (Figure 5a). The percentage of chickens at drinkers changed according to feeding behaviour (Figure 5b): AL chickens drank concurrently with feed distribution, reaching a peak after 5 min (5.9%), whereas FR chickens started to drink only 5 min after feed distribution, showing a progressive and significant increase in the percentage of chickens at drinkers 15 minutes after the feed was distributed. The percentage of standing (16.9% vs. 6.4%) birds 10 min before feed distribution (Figure 5c) was higher in AL compared to FR chickens whereas 5 min after feed distribution the differences between the two groups was reversed (9.9% vs. 13.0%) (*p* < 0.001). The percentage of inactive animals, sitting or lying on the litter (Figure 5d), was similar in the two groups 10 min before (very high, >70%) and at the time of feed distribution (very low, <10%), but differed 5 min after, with higher values reached in AL chickens (59.1% vs. 0.91%; *p* < 0.001). In FR birds, the percentage of inactive animals started to increase after 10 and 15 min following feed distribution. At the time of feed distribution, a higher percentage of AL birds was also seen walking compared to FR birds Figure 5e). Finally, during the 10 min-period before feed distribution, chickens showed comfort behaviours (Figure 5g), with a higher percentage in FR chickens than in AL ones (6.3% vs. 1.3%; *p* < 0.001). At the time of feed distribution, no comfort behaviours were observed until after 5 min and then only in AL animals.

### 3.3. Corticosterone Changes in Plasma and Faeces

Corticosterone content gradually decreased with age both in plasma (from 2.67 ng/mL at 14 d of age to 2.25, 1.54 and 1.31 ng/mL at 21 d, 28 d and 35 d; SEM: 0.347 ng/mL; *p* < 0.01) and in faeces (from 18.0 ng/g at 14 d of age to 13.9, 11.7, 10.7, and 10.3 ng/g; SEM: 0.928 ng/g; *p* < 0.001) (data not reported in tables). Moreover, corticosterone tended to be higher in the faeces of FR compared to AL birds (13.6 vs. 12.2 ng/g; *p* ≤ 0.10) and in high compared to standard breast yield groups (13.7 vs. 12.1 ng/g; *p* < 0.10) (data not reported in tables). No significant effect of sex was measured.

**Figure 6:**
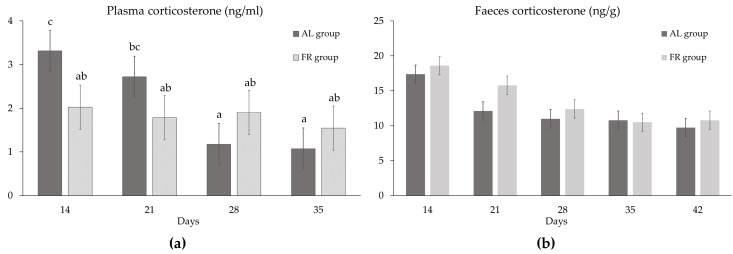
Corticosterone contents (LS means±SEM) at different ages in plasma (Probability of the interaction feeding system x age, *p* < 0.05) (**a**) and faeces (Probability of the interaction feeding system x age, *p* > 0.05) (**b**) of chickens fed ad libitum (AL) or submitted to restriction (FR). Different letters stand for significant differences among LS means (Bonferroni t test).

Finally, a significant interaction was observed between the age and the feeding system for plasma corticosterone (*p* ≤ 0.05), but not for faeces corticosterone (Figure 6). In fact, the content of plasma corticosterone was significantly higher in AL chickens compared to FR chickens at 14 d and 21 d of age, whereas no significant difference was observed between AL and FR chickens at the last two recordings or among FR chickens at the different ages (Figure 6a).

## 4. Discussion

### 4.1. Effect of the Feeding System

In the present trial, feed restriction decreased final live weight (3194 vs. 3142 g; *p ≤* 0.01) and feed conversion (1.57 vs. 1.60; *p ≤* 0.01) compared to ad libitum feeding [21]. Moreover, it significantly increased activity consistently with previous observations in broiler chickens [21] and breeding layers [22,23] when quantitative feed restriction was compared with qualitative feed restriction (i.e., low energy and low protein diets) during the period of feed restriction. The interaction between the feeding system and the age showed that the effect of feed restriction on chicken activity lasted beyond the feed restriction period even if the magnitude of differences between FR and AL groups, in terms of birds standing and laying/sitting, decreased with increasing age. On the other hand, the percentage of chickens at feeders remained higher in birds subjected to early feed restriction for some days beyond feed restriction, which may be explained by a continuing sense of hunger maintained up to 25 d but disappeared by 35 days of age.

In breeding birds, other authors have also observed that chickens increased their pecking activity towards objects inside the pen in the case of a quantitative restriction rather than a qualitative one, which was considered a sign of higher stress conditions [22,23]. Indeed, previous studies have also observed a greater plasma corticosterone concentration in feed restricted birds compared to ad libitum ones indicating a greater stress on the former [2,24].

Nevertheless, under the conditions of the present study, the feed restriction rate used from 13 d to 21 d of age (80% of the ad libitum intake) likely was not so strict to severely challenge chicken welfare. In fact, the concentration of corticosterone in plasma did not change, whereas it only tended to be higher (*p* < 0.10) in the faeces of feed restricted animals compared to those fed ad libitum. This mild increase may indicate an increase in stress in feed restricted animals and is in agreement with behavioural observations and, possibly, a chickens’ adrenergic reactivity [25]. Animals increased their activity when restricted, but did not show any aggressive attitudes towards other birds. On the other hand, when restricted, the percentage of animals pecking in feeders increased and comfort behaviours decreased.

### 4.2. Effect of Sex

At the end of the trial, males showed higher final live weight (3492 vs. 2845 g) and lower feed conversion (1.54 vs. 1.63) than females (*p ≤* 0.001) [16], whereas body size differences between males and females are well known. On the other hand, little information is available regarding behavioural differences according to sex, which may affect animal welfare in broilers. As measured in this study in terms of chickens pecking other chickens, Millman et al. [7] reported that male broilers were more aggressive than females and reported that males were aggressive towards females when placed together. Bokkers and Koene [6] found that male broilers walk faster to a food reward than females, especially when restriction fed, which is consistent with our observations of a higher percentage of males feeding at least during the first part of the trial (until 25 d). On the other hand, the lower percentage of walking males we recorded at the late observations at 39 d and 45 d of age is likely related to their higher body size [16] and lower walking ability/comfort [26] compared to females.

### 4.3. Effect of Genotype

In this study, standard broilers were heavier (3270 vs. 3139 g; *p* ≤ 0.001) and showed lower feed conversion (1.56 vs. 1.61; *p ≤* 0.001) than the high breast yield chickens [16], whereas the changes in animal behaviour according to genotype showed a significant trend in the percentage of standing animals (higher in high-breast chickens) and in the percentage of animals pecking the floor (higher in standard-breast chickens). On the other hand, the significant interactions observed between the age of the animals and genotype are less relevant from a quantitative and qualitative point of view. A larger effect of genotype on behaviour would have likely occurred if slow-growing genotypes would have been compared to fast-growing genotypes because of differences in growth rate, in final live weight and, thus, their walking ability/comfort.

Nevertheless, there was a tendency for an increase in the corticosterone content in the faeces of animals with a high breast yield compared to those with standard yield. It could be hypothesized that this result was related to differences in physiology rather than in the stress conditions between the two genotypes. As reviewed by van Krimpen and de Jong [27], food ingestion is regulated by physical (emptying/filling of the digestive tract and crop) and chemostatic mechanisms, which include plasma glucose levels and gastro-intestinal hormones (cholecystokinin and neuropeptide Y) that are produced when feed or specific nutrients are available in the intestinal tract, as well as levels of plasma corticosterone and neurotransmitters (dopamine and serotonin). In chickens selected for fast growth, overfeeding is usually observed whereas relationships between the control of food intake and the energy requirements and balance have not yet been fully elucidated [28]. In our study the high-breast chickens, that have higher faeces corticosterone, showed a higher feed intake compared to standard breast chickens in the first period [16]. According to van Krimpen and de Jong [27] the role of cholecystokinin, neuropeptide Y, glucose and corticosterone in determining hunger, satiety and, therefore, the level of food intake, is not completely clear and more research is needed.

### 4.4. Effect of Age

Generally speaking, and consistent with our results, other authors have also described a reduction in activity starting from the third week of age in chickens selected for rapid growth [21,29,30]. This could be due to the increase of weight or to the increase of the chicken’s thermoregulatory ability where increased metabolism with growth rate as a function of age can provoke a consequent increase in heat production [21]. On the other hand, the decrease in activity could be the result of greater difficulty in walking due to increasing body weight [26]. Bokkers and Koene [6] verified using motivation tests that the reduction of activity is correlated to the greater difficulty of movement rather than to a reduction in the motivation to move. Also, the reduction in the percentage of animals pecking the litter during the first three weeks of observation could be explained by both the reduction of animal explorative behaviour with age in the absence of novel stimuli [31] and the reduction of feed wasted by animals (feed taken out of the feeders).

Regarding physiological stress indicators, the reduction of plasma corticosterone with increasing bird age was also reported in other studies on broiler breeders [22]. It could be also hypothesized a relationship with mechanisms controlling voluntary feed intake: during the first weeks of age the ingestion of food may be conditioned more by physical mechanisms (filling of the digestive system) whereas the mechanism of chemostatic regulation of appetite changes with age which can affect the corticosterone levels [27,28,32]. In the present study, the reduction in corticosterone concentration with increasing animal age was also confirmed by analysis of faeces.

On the other hand, Sandilands et al. [23] did not observe any significant differences in the plasma concentration of corticosterone measured at different ages (41, 82 and 125 days of age) in pullets destined for reproduction and subjected to feed restriction (quantitative or qualitative). In this case, the lack of differences in plasma corticosterone in chickens subjected to continuous feed restriction may be explained by the continuing sense of hunger.

## 5. Conclusions

Several management and ontogenetic factors contribute to broiler chicken welfare and affect their behaviour during rearing. In the present trial, a moderate feed restriction stimulated activity during and for some time beyond the restriction phase with a small effect on stress condition despite feeding and comfort behaviour were affected during restriction. Differences in behaviour between the two fast-growing genotypes tested were limited in quantity and quality. Finally, the effects of both sex and age were related to chicken size so that heavier animals moved less when their walking ability/comfort decreased.

## Figures and Tables

**Table 1:** Ethogram of mutually exclusive behaviours.

Behaviour	Description
Feeding	Chickens pecking in feeders
Drinking	Chickens at drinkers
Standing	Stand up
Sitting/lying	Sitting or lie down
Walking	Walking or running with no other discernible activity
Pecking floor	Pecking the floor (including feeding fodder)
Pecking other bird	Pecking any body part of other birds
Comfort	Other comfort behaviours, such as preening, scratching or wing stretching

**Table 2:** Percentage of chickens per pen (LS means ± SEM) performing a behaviour: effect of feeding system, genotype, sex and age.

	Feeding System (F)		Genotype (T)		Sex (S)		*p*-Value ^1^			
Behaviours	Ad libitum	Restricted	Standard	High	Females	Males	F	T	S	Age
Feeding	7.51 ± 0.18	8.17 ± 0.19	7.83 ± 0.19	7.84 ± 0.19	7.69 ± 0.18	7.98 ± 0.19	0.011	0.978	0.277	<0.001
Drinking	3.51 ± 0.11	3.72 ± 0.11	3.64 ± 0.11	3.59 ± 0.11	3.48 ± 0.11	3.75 ± 0.11	0.181	0.750	0.089	<0.001
Standing	9.84 ± 0.20	11.71 ± 0.23	10.22 ± 0.20	11.27 ± 0.22	10.92 ± 0.22	10.54 ± 0.21	<0.001	<0.001	<0.001	<0.001
Sitting/lying	67.03 ± 0.38	64.14 ± 0.37	65.99 ± 0.38	65.15 ± 0.37	65.47 ± 0.38	65.67 ± 0.38	<0.001	0.118	0.700	<0.001
Walking	1.10 ± 0.06	1.14 ± 0.06	1.15 ± 0.06	1.09 ± 0.06	1.18 ± 0.07	1.06 ± 0.06	0.682	0.491	0.182	<0.001
Pecking floor	0.15 ± 0.02	0.16 ± 0.02	0.20 ± 0.03	0.12 ± 0.02	0.14 ± 0.02	0.18 ± 0.03	0.807	0.008	0.155	<0.001
Pecking bird	0.10 ± 0.82	0.06 ± 0.63	0.10 ± 0.76	0.07 ± 0.71	0.05 ± 0.57	0.11 ± 0.86	<0.001	0.990	<0.001	<0.001
Comfort	5.94 ± 0.14	5.80 ± 0.14	5.88 ± 0.14	5.86 ± 0.14	6.06 ± 0.14	5.68 ± 0.13	0.499	0.891	0.049	<0.001

^1^*p*-values of all interactions are given in Table S1.

**Table 3:** Percentage of chickens per pen (LS means ± SEM) performing a behaviour at 18 d: effect of time with respect to feed administration.

Behaviours ^1^	Time with Respect to Feed Administration (F)					*p*-value ^2^
	−10 min	0	+5 min	+10 min	+15 min	
Feeding	0.14 ^a^ ± 0.41	33.08 ^c^ ± 2.28	32.26 ^c^ ± 2.29	21.20 ^b^ ± 1.84	11.14 ^a^ ± 1.08	≤0.001
Drinking	0.84 ^a^ ± 0.27	0.00 ^a^ ± 0.35	3.58 ^b^ ± 0.72	4.35 ^bc^ ± 0.86	6.97 ^c^ ± 1.07	≤0.001
Standing	10.44 ^b^ ± 0.92	27.28 ^c^ ± 1.71	11.33 ^b^ ± 0.92	5.08 ^a^ ± 0.58	11.48 ^b^ ± 1.01	≤0.001
Sitting/lying	74.40 ^d^ ± 3.41	8.87 ^a^ ± 0.81	7.33 ^a^ ± 1.38	27.81 ^b^ ± 1.85	43.45 ^c^ ± 2.33	≤0.001
Walking	0.83 ^a^ ± 0.46	6.72 ^b^ ± 1.95	3.66 ^ab^ ± 1.11	1.64 ^a^ ± 0.56	0.00 ^a^ ± 0.32	≤0.001

^1^ Behaviors (pecking the floor, pecking other birds) which data were not suitable for statistical analyses because of their low frequency of occurrence are not included in the table. ^2^ All *p*-values of the effects tested in the model (feeding system, genotype, sex, time with respect to feed distribution and their interactions) are given in Table S2. ^a,b,c^Means with different superscript letters significantly differ.

## Supplementary Materials

The following are available online at https://www.mdpi.com/2076-2615/10/5/830/s1, Table S1: Probability of the effect of the interactions between age (A, 11, 18, 25, 32, 39 and 45 d), feeding system (F, ad libitum vs. restricted), genotype (T, standard vs. high breast yield), and sex (S, females vs. males) on the percentage of chickens per pen performing a behaviour. Table S2: Probability of the effect of time (minutes, M) with respects to feed distribution (−10, 0, +5, +10, +15 min), feeding system (F, ad libitum vs. restricted), genotype (T, standard vs. high breast yield), and sex (S, females vs. males) and their interactions on the percentage of chickens per pen performing a behaviour at 18 d. Figure S1: Percentage of chickens per pen (LS means ± SEM) performing a behaviour; average of recordings at 11, 18, 25, 32, 39 and 45 d of age; significant interactions between feeding system × genotype (a), and between genotype × sex (b, c).
