# Supplementary material for: Effect of Feed Restriction on the Behaviour and Welfare of Broiler Chickens
Source: Animals (Basel). 2020 May 11;10(5):830. doi: 10.3390/ani10050830 (PMC7278418; doi:10.3390/ani10050830)
Supplement: Supplementary file 1 [file animals-10-00830-s001.pdf]

Supplementary materials

Table S1. Probability of the effect of the interactions between age (A, 11, 18, 25, 32, 39 and 45 d), feeding system (F, ad libitum *vs*. restricted), genotype (T, standard *vs*. high breast yield), and sex (F, females *vs*. males) on the percentage of chickens per pen performing a behaviour.

**Behaviours[1]**	**A × F**	**A × T**	**A × S**	**F × T**	**F × S**	**T × S**
Feeding	<0.001	<0.001	<0.001	0.130	0.285	0.610
Drinking	<0.001	0.040	<0.001	0.144	0.087	0.016
Standing	<0.001	<0.001	<0.001	0.379	0.220	0.069
Sitting/lying	<0.001	<0.01	<0.001	0.054	0.158	0.043
Walking	<0.001	<0.001	<0.001	0.295	0.418	0.286
Pecking floor	<0.001	<0.001	0.001	0.017	0.506	0.143
Comfort	<0.001	<0.001	<0.001	0.629	0.465	0.142

[1]*p*-value is missing for behaviours (pecking other bird) which data were not suitable for statistical analyses because of their low frequency of occurrence.

Table S2. Probability of the effect of time (minutes, M) with respects to feed distribution (-10, 0, +5, +10, +15 min), feeding system (F, ad libitum vs. restricted), genotype (T, standard vs. high breast yield), and sex (S, females vs. males) and their interactions on the percentage of chickens per pen performing a behaviour at 18 d.

**Behaviours[1]**	**M**	**F**	**T**	**S**	**F × M**	**F × T**	**F × S**	**T × S**
Feeding	<0.001	0.933	0.165	0.720	<0.001	0.300	0.635	0.324
Drinking	<0.001	0.988	0.066	0.790	<0.001	0.029	0.953	0.324
Standing	<0.001	0.193	<0.001	0.619	<0.001	<0.001	0.132	0.074
Sitting/lying	<0.001	<0.001	0.695	0.286	<0.001	0.004	0.960	0.200
Walking	<0.001	0.996	0.738	0.096	<0.001	0.424	0.815	0.117
Comfort	<0.001	<0.001	0.514	0.125	<0.001	0.637	0.001	0.998

[1]*p*-value is missing for behaviours (pecking the floor, pecking other bird) which data were not suitable for statistical analyses because of their low frequency of occurrence.

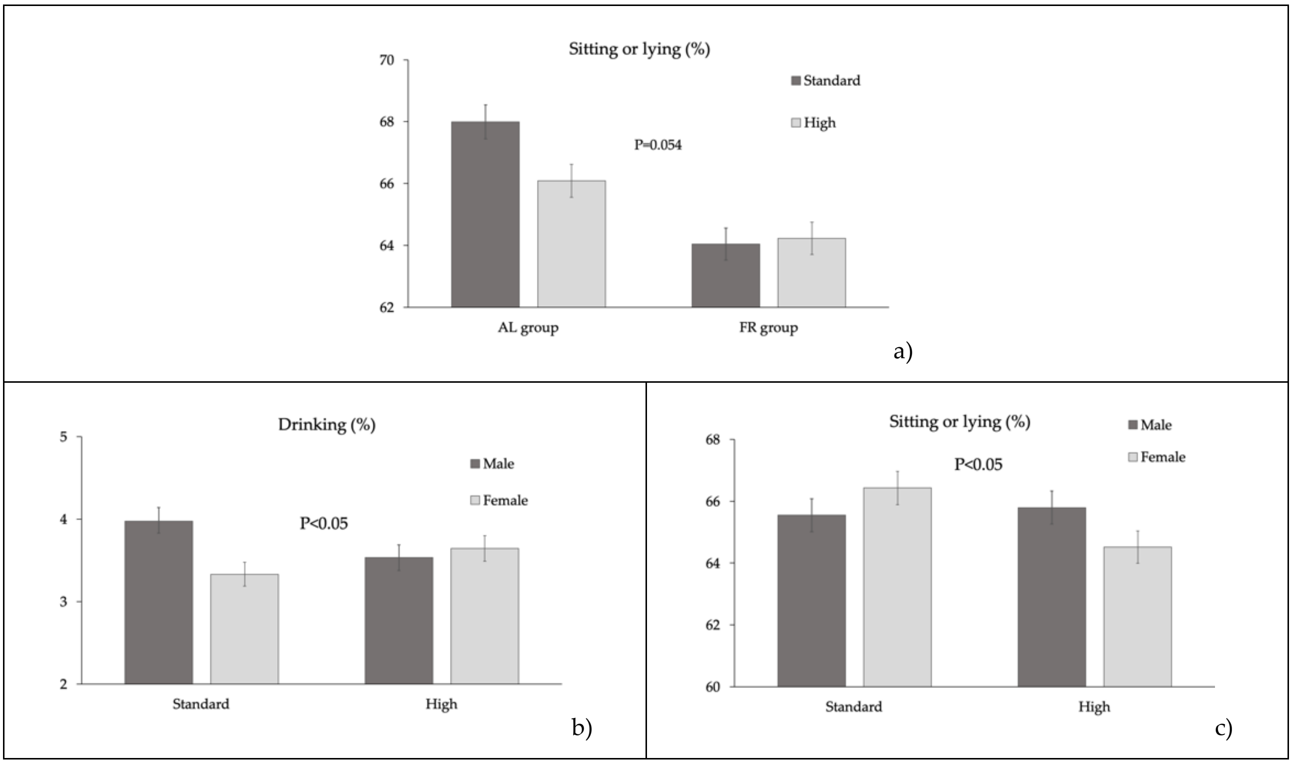

Figure S1. Percentage of chickens per pen (LS means ± SEM) performing a behaviour; average of recordings at 11, 18, 25, 32, 39 and 45 d of age: significant interactions between feeding system × genotype (a) and between genotype × sex (b, c).
